# The non-canonical functions of the heme oxygenases

**DOI:** 10.18632/oncotarget.11923

**Published:** 2016-09-09

**Authors:** Luca Vanella, Ignazio Barbagallo, Daniele Tibullo, Stefano Forte, Agata Zappalà, Giovanni Li Volti

**Affiliations:** ^1^ Department of Drug Sciences, University of Catania, Catania, Italy; ^2^ Division of Haematology, AOU “Policlinico - Vittorio Emanuele”, University of Catania, Catania, Italy; ^3^ Department of Biomedical and Biotechnological Sciences, University of Catania, Catania, Italy; ^4^ Istituto Oncologico del Mediterraneo Ricerca srl Viagrande, Catania, Italy; ^5^ EuroMediterranean Institute of Science and Technology, Palermo, Italy

**Keywords:** heme oxygenase, nuclear translocation, protein-protein interaction, non-canonical functions, extracellular space

## Abstract

Heme oxygenase (HO) isoforms catalyze the conversion of heme to carbon monoxide (CO) and biliverdin with a concurrent release of iron, which can drive the synthesis of ferritin for iron sequestration. Most of the studies so far were directed at evaluating the protective effect of these enzymes because of their ability to generate antioxidant and antiapoptotic molecules such as CO and bilirubin. Recent evidences are suggesting that HO may possess other important physiological functions, which are not related to its enzymatic activity and for which we would like to introduce for the first time the term “non canonical functions”. Recent evidence suggest that both HO isoforms may form protein-protein interactions (i.e. cytochrome P450, adiponectin, CD91) thus serving as chaperone-like protein. In addition, truncated HO-1 isoform was localized in the nuclear compartment under certain experimental conditions (i.e. excitotoxicity, hypoxia) regulating the activity of important nuclear transcription factors (i.e. Nrf2) and DNA repair. In the present review, we discuss three potential signaling mechanisms that we refer to as the non-canonical functions of the HO isoforms: protein-protein interaction, intracellular compartmentalization, and extracellular secretion. The aim of the present review is to describe each of this mechanism and all the aspects warranting additional studies in order to unravel all the functions of the HO system.

## INTRODUCTION

**Figure 1:**
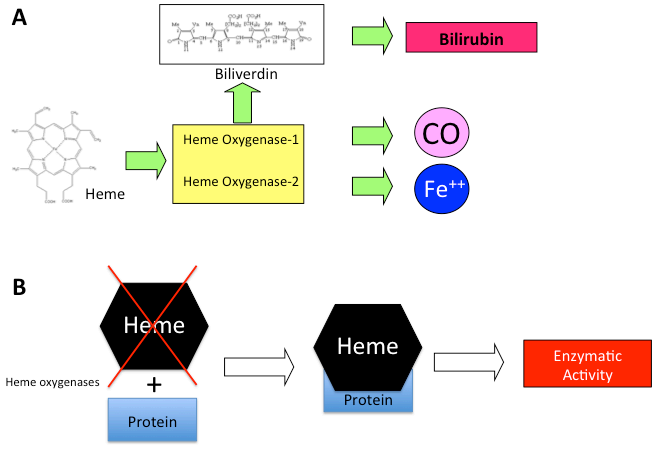
**A.** Schematic representation of enzymatic reaction catalyzed by HO isoforms and **B**. mechanism of heme dependent protein regulation.

**Figure 2:**
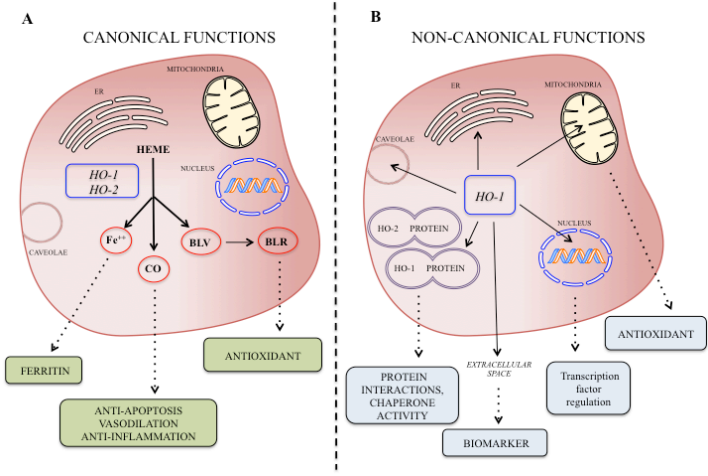
**A.** Canonical and non-canonical **B.** functions of the HO isoforms at a glance and possible biological significance of their byproducts.

Heme oxygenases catalyze the degradation of heme into biliverdin, carbon monoxide (CO) and ferric iron [1-5] (Figure 1A). Heme functions as the prosthetic group in hemoproteins, e.g., nitric oxide synthase, cyclooxygenases, soluble guanylate cyclase, cytochrome P450, peroxidase, and catalase and since HO is the sole physiological pathway of heme degradation. It consequently plays a critical role in the regulation of cellular heme-dependent enzyme levels [6-10] (Figure 1B). To date, two HO isoforms have been shown to be catalytically active in heme degradation and each is encoded by a different gene [2, 11]. HO-1 is expressed at low levels under basal conditions and it is induced by polyphenols [12-18], statins [19], metals [20-23] and a variety of stimuli such as inflammation, oxidative stress, hyperoxia, hypoxia and trauma [24-30]. Such upregulation represents an intrinsic defense mechanism to maintain cellular homeostasis and enhance cell survival [31-34]. In particular, HO-1 is considered to play a major role as an essential survival factor, protecting against chemotherapy-induced reactive oxygen species (ROS) increase [27, 35-39]. Most of the studies so far were directed at evaluating the protective effect of these enzymes because of their ability to generate antioxidant and antiapoptotic molecules such as CO and bilirubin [40-47]. In contrast, HO-2 is responsible for the most HO constitutive activity [48-51]. Interestingly, recent evidence suggests that HO may possess other important physiological functions, which are not related to its enzymatic activity and for which we would like to introduce for the first time the term “non canonical functions” (Figure 2). In particular, we discuss three potential signaling mechanisms that we refer to as the non-canonical functions of the HO isoforms: protein-protein interaction, intracellular compartmentalization, and extracellular secretion. The aim of the present review is to describe each of this mechanism and the aspects warranting additional studies in order to unravel all the functions of the HO system.

## PROTEIN-PROTEIN INTERACTION

The first paper reporting the possibility that HO isoforms may form protein-protein interaction was suggested in the elegant description of HO-1 purification where a 68-kDa protein was identified [4]. It is now known that the molecular mass of HO-1 is 32 and HO-2 36 kDa. Therefore, it is conceivable that a complex of the two proteins was initially isolated under partially denaturing conditions. On the basis of this simple observation, Weng Y. et al [52] showed an interaction between HO-2 and HO-2 and demonstrated the effect of the HO-1/HO-2 protein complex on HO activity. The authors elegantly concluded that this interaction serves to limit HO activity in certain tissues where the two co-enzymes co-localize and such negative regulation of HO activity may be important to ensure a cytoprotective range of HO expression. However this intriguing evidence and possible non-canonical functions of HO isoforms remained unexplored for several years until when our research group was able to demonstrate the presence of HO-1 isoform in the extracellular compartment (i.e. human milk) [53]. The presence of the protein in such biological fluid was not particularly surprising given the apocrine nature of the mammary gland. However, since the enzymatic activity was undetectable in human milk, we were wondering on the possible biological significance of such protein in the extracellular compartment. To this regard, basing on the HO-1 amino acidic sequence homologies with Hsp70, we identified CD91 as a possible interactor of HO-1 in the extracellular space. To this regard, PatchDock molecular docking algorithm and FireDock analysis showed that the charged and polar residues observed on HO-1 are Glu63, Tyr78, Glu81, Glu82, His84, Lys86, Glu90, Gln91 and Gln102. The charged and polar residues observed on CD91 in the region are Arg571, Thr576, Thr536, Arg553, Trp556 and Ser565. Hydrogen bond interactions were observed between Glu90 and Gly571, between Glu63 and Thr576, and between Gln102 and Thr536. Furthermore, hydrophobic-hydrophobic interactions were observed between Tyr55, Val59 (HO-1) and Val535 (CD91). Salt bridges were also observed between Lys177 (HO-1) and Glu332 (CD91) outside of this region. Our successive studies also showed the possibility to form protein-protein interaction also for the HO-2 isoform. In particular, this observation derived again from a casual observation of HO-2 −/− animals exhibiting a metabolic syndrome phenotype and reduced circulating levels of adiponectin [54]. Our data showed that pharmacological upregulation of HO-1 rescued such phenotype and restored adiponectin levels. This adipokyne is formed in the endoplasmic reticulum and requires specific chaperone activity in order to maturate and be secreted in the extracellular space in its active form [55]. Given the endoplasmic reticulum localization of HO-2 and the possibility that it may form protein-protein interaction with other proteins we decided to test the hypothesis that HO-2 may serve as a chaperone for physiological secretion of adiponectin from the endoplasmic reticulum. In particular, our in silico analysis showed two hypothetical symmetrical binding regions making contact with two different regions of adiponectin were identified. Multiple structural motifs appear to be involved in both the recognition and binding process between HO-2 and adiponectin. In particular, an extended and structured area of 13 amino acids (a.a. 235-247) in HO-2 seems to interact with a specific sequence of adiponectin (a.a. 238-246). Interestingly one single Arginine residue on the HO was found in the two hypothetical contact regions. These results were further validated *in vivo* by using the Bacteriomatch two-hybrid system.

Similar results were also obtained for HO-2 isoform by Spencer AL et al. [56] by Fluorescence Resonance Energy Transfer. In particular, the authors showed that this protein may form a protein-protein interaction with cytochrome P450 reductase leading to the formation of a dynamic ensemble of complex(es) that precede formation of the productive electron transfer complex. Finally, Williams SE et al showed that HO-2 is part of the calcium-sensitive potassium (BK) channels complex and enhances channel activity in normoxia [57]. In particular, the authors showed that inhibition of BK channels by hypoxia was dependent on HO-2 expression and was augmented by HO-2 stimulation.

## INTRACELLULAR COMPARTMENTA-LIZATION OF HEME OXYGENASES

**Figure 3:**
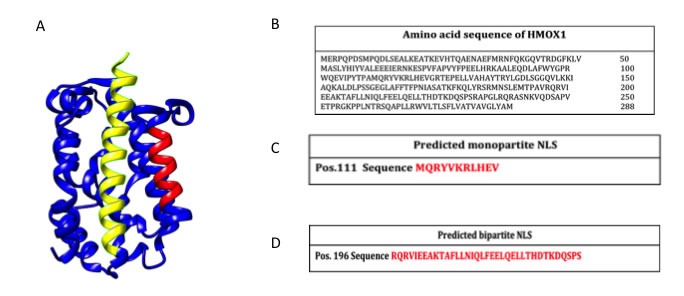
**A.** 3D structure of HO-1 (Structural Biology Knowledgebase: 3TGM, UniProtKB AC: P09601). In red the predicted monopartite Nuclear Localization Signal (NLS) and in yellow the predicted bipartite NLS. Molecular graphics and analyses were performed with the ChimeraX package; **B.** Amino acid sequence of HO-1 (GenBank: CAG30391.1); **C.** Prediction of monopartite NLSs specific of HMOX1 (cNLS Mapper tool); **D.** Prediction of bipartite NLSs of HMOX1 (cNLS Mapper tool).

**Figure 4:**
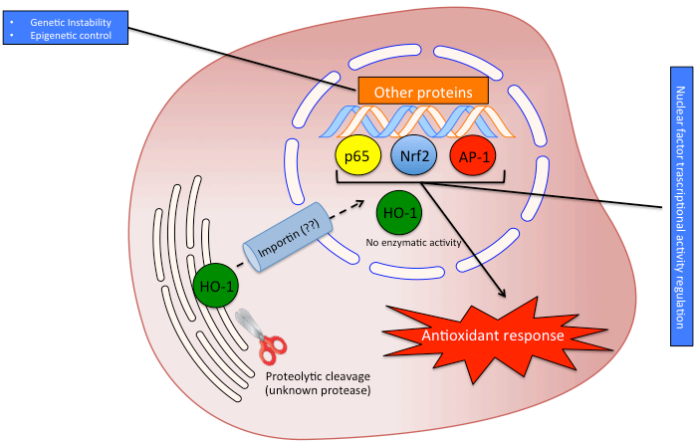
Possible significance of HO-1 nuclear translocation The proteolytic cleavage allows the translocation of HO-1 into the nucleus probably by interaction with importin. In the nucleus HO-1 loses enzyme activity and regulates the transcriptional factors activity interacting with p65, AP-1 and Nrf2.

**Figure 5:**
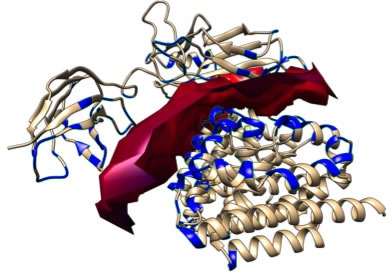
Docking simulation for p65/HO-1 interaction According to the predicted molecular complex structure, p65 (upper structure) and HO-1 (lower structure) binding is defined by and extended surface of molecular complementarity. Interaction surface is represented by solid red area while residues involved in protein contacts are represented in blue. The local estimated energy of the binding is -31.65 kcal/mol.

As above mentioned the HO isoforms were identified and localized in the endoplasmic reticulum where they exert their enzymatic function. However, later studies were devoted at investigating the subcellular compartmentalization of these two isoforms [58]. As far as concerning the HO-2 isoform, no studies so far identified the presence of this enzyme in other cellular compartment rather than the endoplasmic reticulum. On the contrary, HO-1 isoform was found to be compartmentalized (i.e. nuclei, mitochondria, caveolae) intracellularly under various experimental conditions and different cell types. The most studied and fascinating aspects of the non-canonical functions of HO-1 are related to nuclear translocation. Such possibility is substantiated by bioinformatic analysis demonstrating the nuclear import sequence into the amino acidic sequence of HO-1 (Figure 3A-3D). Successive studies demonstrated nuclear translocation under various experimental conditions. In particular, Suttner DM et al. [59] showed that HO-1 migrated into the nuclear compartment following oxygen toxicity in lung cells and such translocation may account for the regulation of cytoprotective pathways. Similarly, we also demonstrated that excitotoxic injury leads to a significant increase of HO-1 protein expression in primary astroglial cell cultures and a concomitant nuclear translocation of this protein [60]. However, at that time we were not able to identify any possible role of nuclear HO-1 and we proposed only that nuclear HO-1 may still possess the ability to bind heme and may therefore serve as a regulator of heme dependent transcription factor [61]. Since then, several other reports evaluated the presence of HO-1 into the nuclear compartment. In particular, Lin Q et al [62] elegantly showed that HO-1 translocate into the nuclear compartment under hypoxic condition and this is associated with increased activation of antioxidant responsive promoter and activation of transcription factors such as AP-1 and NFkB which are also known transcription factors of HO-1 itself [63-65] (Figure 4). These results were also confirmed by the same group in a transgenic animal model of lung injury following hyperoxia [66] in which the authors showed that nuclear translocation overexpression inhibits repair from hyperoxic lung injury by inhibiting DNA repair, which may predispose the lung to later malignant transformation. Consistently with these data, our docking analysis showed that HO-1 might interact with p65 subunit of NFkB (Figure 5). According to simulations p65 interacting surface seems not to involve DNA binding domains suggesting that the inhibitory control may be exerted by allosteric control. Successive reports showed that nuclear localization of HO-1 was associated with cancer stadiation or chemoresistance. As far as concern clinical stadiation of cancer, Gandini NA et al [67] showed that nuclear HO-1 increases with tumor progression in a mouse model of squamous cell carcinoma and in human head and neck squamous cell carcinoma. Interestingly, the same authors showed that no association of HO-1 nuclear localization with glioblastoma patients survival was detected [68] thus suggesting that nuclear translocation of this protein occurs only under certain specific pathological conditions. Consistently with these results Wegiel B et al. [69] also showed that nuclear HO-1 exhibits reduced enzymatic activity and correlates with poorer prognosis in prostate cancer. As far as concern chemoresistance our research group showed that nuclear translocation confers resistance to imatinib in chronic myeloid leukemia cells [70]. Interestingly, we showed that inhibition of nuclear HO-1 translocation by E64d, a cysteine protease inhibitor, restores sensitivity to imatinib, whereas HO byproducts CO or bilirubin had no effects. Similarly, we have recently shown that HO-1 nuclear translocation is also associated to chemoresistance to Bortezomib, a proteasome inhibitor, in various multiple myeloma cell lines [71]. Interestingly, we showed that nuclear translocation of HO-1 was associated to genetic instability thus suggesting that other functions, beside interaction with nuclear transcription factors, could be associated with nuclear HO-1 (Figure 5). Therefore, it becomes of great importance to identify the precise mechanisms underlying HO-1 cleavage allowing its possible nuclear localization in order to overcome chemoresistance. To this regard, the signal peptide peptidase (SPP) catalyzes the intramembrane cleavage of HO-1 allowing nuclear translocation and promoting cancer cell proliferation and invasion independently from its enzymatic activity [72].

Other subcellular localizations have been described for HO-1 and may be related to its non-canonical functions. In particular, Converso DP et al. [73] demonstrated for the first time the localization of HO-1 protein in mitochondria suggesting its important biological roles in regulating mitochondrial heme protein turnover and in protecting against conditions such as hypoxia, neurodegenerative diseases, or sepsis, in which substantially increased mitochondrial nitric oxide and oxidant production have been implicated. Similar results were obtained by Slebos DJ et al. [74] showing that HO-1 localized to mitochondria in a primary culture of human small airway epithelial cells following cigarette smoke extract exposure. Interestingly, the authors showed that such translocation was accompanied by a significant increase of the HO mitochondrial activity. These results were confirmed by Bindu S et al. [75] showing that mitochondrial translocation of HO-1 also resulted in time-dependent inhibition of apoptosis during gastric mucosal injury following indomethacin treatment. The mitochondrial significance of HO-1 was further supported by the interesting observations of Bolisetty S et al [76], which were able to target specifically renal epithelial cell mitochondria with HO-1 protein. In these set of experiments the authors showed that specific mitochondrially targeted HO-1 under acute pathological conditions may have beneficial effects, but the selective advantage of long-term expression is constrained by a negative impact on the synthesis of heme-containing mitochondrial proteins. This latter observation was further confirmed by Bansal L et al [77] showing that cells expressing mitochondria targeted HO-1 exhibited higher ROS production leading also to increased autophagy and reduction of cytochrome c oxidase activity. Finally, HO-1 was also demonstrated in caveolae, the small flask-shaped and detergent insoluble invaginations in plasma membrane and are implicated to function in the vesicular transport processes and the transduction of receptor generated signals. To this regard Jung NH et al. [78] showed that HO-1 is localized in caveolae of mouse mesangial cells where it may co-localize with important proteins such as caveolin-1 and caveolin-2. In addition, Wang XM et al showed that such translocation is dependent on p38MAPK and it may regulate the interaction between caveolin-1 and Toll Like Receptor-4 [79].

## THE HEME OXYGENASES IN THE EXTRACELLULAR SPACE

Several reports evaluated so far the presence of HO-1 in extracellular compartments and biological fluids thus suggesting that HO-1 may serve as a possible biomarker of disease [80, 81] or may play additional roles in the extracellular space as a receptor ligand [82]. Interestingly, no reports reported the presence of HO-2 in such compartments thus suggesting that the presence of HO-1 is not the results of cell necrosis and passive release in extracellular compartments but could be related to a still unknown specific mechanism of secretion. In the following sections we will therefore evaluate the significance only of HO-1 in various biological fluids.

### HO-1 in plasma and serum

Most of the reports evaluating the presence of HO-1 in extracellular space refer to serum or plasma. In particular, Eide IP et al. [83] showed that serum HO-1 levels were significantly higher among pre-eclampsia patients compared to controls supporting the role of oxidative stress and excessive maternal inflammatory response in the pathogenesis of pre-eclampsia. Other clinical studies reported the increase of serum HO-1 under other pathological conditions such as Alzheimer disease. In particular, Mueller C et al [84] showed that serum HO-1 among other proteins is increased in Alzheimer's disease and such levels correlated to cognition impairment grade. Similarly plasma HO-1 is increased in patients resuscitated from out-of-hospital cardiac arrest [85] or suffering from peripheral artery disease [80]. These results were further confirmed in an animal model of lung injury induced by Ischemia/reperfusion [86] in which the authors showed increased serum HO-1 during the 3h observation period.

As far as concern the possible release mechanism(s) of HO-1 in serum or plasma, no data are so far available. However, it is possible that HO-1 requires proteolytic cleavage in order to be secreted. To this regard, Zager RA et al. [87] showed that plasma HO-1 is increased in patients suffering acute kidney injury and interestingly the western blot showed a 16Kda band rather than the canonical 32Kda of the full-length protein. Taken all together, these data suggest that HO-1 is increased in plasma or serum in those all conditions in which oxidative stress is increased. Therefore, even though the release of HO-1 in the extracellular space may be of some biological importance, its possible use as a potential biomarker for a particular disease could be limited by its specificity. Furthermore, the evidence that liraglutide treatment resulted in a significant reduction of plasma HO-1 levels in type-2 diabetes mellitus patients supports the idea that extracellular HO-1 should be considered as an active secretory mechanism. Furthermore, the hypothesis that plasma HO-1 is the results of an active mechanism of secretion and not the consequence of cell necrosis is supported by previous work showing that HO-1 is increased in patients with acute myocardial infarction independently of cell necrosis biomarkers (i.e troponin and creatine phosphokinase) [25].

### HO-1 in cerebrospinal fluid

Cerebrospinal fluid is often used for the evaluating central nervous system specific markers and as a diagnostic routine (i.e. multiple sclerosis) [88]. To this regard, the biological significance of HO-1 in such biological fluid could be of particular interest. To this regard, Schipper HM et al [89] showed that HO-1 protein is decreased of cerebrospinal fluid of Alzheimer's disease patients. On the contrary, HO-1 is increased in cerebrospinal fluid from infants and children after severe traumatic injury [90-92]. Similarly, a successive study also showed that HO-1 protein is increased in cerebrospinal fluid in patients with Fisher Grade III aneurysmal subarachnoid hemorrhage and this may serve also as an effective outcome indicator in patients with Fisher Grade III aneurysmal subarachnoid hemorrhage [93].

### Conclusions and future perspectives

**Figure 6:**
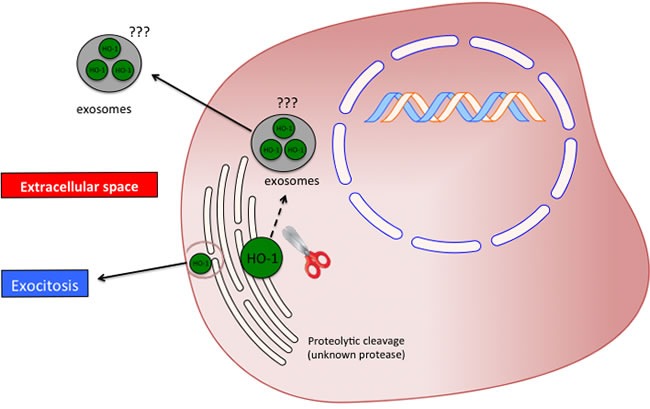
Possible significance and release mechanism of HO-1 in the extracellular space.

Taken all together, the above-mentioned studies suggest that the HO system may possess important biological functions beyond its enzymatic activity. In the present review, we reported what we called the non-canonical function of the heme oxygenases. Three different effects should be included in such class of functions: protein-protein interaction, subcellular compartmentalization and secretion into the extracellular space. However, several issues are still open and warrant future studies. In particular the most important questions needing to be addressed are related to the mechanism(s) underlying proteolytic cleavage of HO-1 allowing the protein to be mobilized from the endoplasmic reticulum. Another important aspect requiring further investigation regards the extracellular release of the protein. As previously discussed, it was reported that the plasma HO-1 is cleaved to a 16 KDa fragment. However, no mechanisms are reported and even hypothesized regarding on how HO-1 is exported in the extracellular compartment. In particular, future studies should be directed in evaluating the presence of HO-1 in exosomes or cellular microvescicles (Figure 6).
